# Differential cardiac geometry during pregnancy in lean versus obese mice

**DOI:** 10.31083/j.rcm2301040

**Published:** 2022-01-20

**Authors:** Kayla Dudick, Chen Che, Robin Shoemaker

**Affiliations:** 1 Department of Dietetics and Human Nutrition, University of Kentucky, Lexington, KY 40506–0054, USA

**Keywords:** Maternal, Pregnancy, Cardiac hypertrophy, Cardiac remodeling, Obesity, Echocardiography

## Abstract

**Background:**

Pregnancy is a sensitive window where factors adversely affecting maternal cardiac health may leave women vulnerable to cardiovascular disease (CVD) later in life. However, it is not clear how cardiac changes during pregnancy influence long-term cardiovascular health. Obesity, an independent risk factor for CVD, promotes adverse cardiac remodeling. Effects of obesity-mediated cardiac remodeling concurrent with physiologic cardiac hypertrophy of pregnancy are not well-studied.

**Methods:**

Female C57BL/6J mice (8 weeks old) were fed a high fat (HF; 60% kcal from fat) or a control low fat (LF; 10% kcal from fat) diet for 8 weeks, then were crossed with male mice to become pregnant (P) or remained non-pregnant (NP) controls. After 18 days, cardiac morphology and function was quantified by echocardiography in LF and HF P and NP mice.

**Results:**

Lean mice had increased left ventricular (LV) mass and LV end-diastolic diameter with pregnancy. In contrast, although LV mass was greater with obesity, it was not augmented with pregnancy in obese mice. Further, pregnant obese mice had decreased LV chamber diameter and increased relative wall thickness compared to lean mice.

**Conclusions:**

We report a differential cardiac geometry during pregnancy in lean versus obese mice in a mouse model of diet-induced obesity. These data suggest obesity during pregnancy promotes concentric remodeling, versus eccentric remodeling in lean mice. Adverse effects of obesity on cardiac remodeling during pregnancy may be an important contributor to subsequent maternal cardiovascular risk.

## 1. Introduction

Epidemiology studies demonstrate that women with a history of pregnancy complications, such as gestational hypertension or diabetes, preterm delivery, or intrauterine growth restriction are at greater risk for mortality from CVD [1]. Cardiac dysfunction and remodeling has been reported in preeclamptic pregnancies [2], suggesting that pregnancy complications may leave women susceptible to subsequent CVD. Obesity is strongly associated with pregnancy complications, with a nearly stepwise increase in the incidence of preterm birth, gestational diabetes, hypertensive disorders, and other high-risk conditions with increasing category of body mass index [3].

Left ventricular (LV) mass is increased in response to pregnancy, but this change is assumed to be transient, and is not associated with cardiac damage. In contrast, obesity-mediated cardiac hypertrophy is pathological, and a prognostic indicator for CVD [4]. Short- and long-term effects of excess adiposity during pregnancy are not well understood. This is important, as adverse cardiac effects during pregnancy may drive future maternal risk for CVD. Limited studies in morbidly obese humans [5,6] and experimental mouse models of metabolic syndrome [7] indicate these conditions impair cardiac adaptation to pregnancy. In a mouse model of diet-induced obesity, we reported augmented LV mass and a different cardiac gene expression profile in mice that had been previously pregnant compared to nulliparous controls [8]. The objective of the current study was to characterize cardiac function and morphology via echocardiography during pregnancy in the same mouse model.

## 2. Methods

### 2.1 Experimental animals

All studies using mice were approved by an Institutional Animal Care and Use Committee (IACUC) at the University of Kentucky and were conducted in accordance with the National Institutes of Health (NIH) Guide for the Care and Use of Laboratory Animals. Female C57BL/6J mice (8 weeks of age; Jackson Laboratory, Bar Harbor, ME, stock # 000664) were randomly assigned to receive, ad libitum, either a high fat (HF, 60% kcal from fat; D12492, Research Diets Inc, New Brunswick, NJ, USA) or a control low fat (LF, 10% kcal from fat; D12450B, Research Diets Inc, New Brunswick, NJ, USA) diet for 8 weeks (n = 30 mice/diet group). Following quantification of body weight (Ohaus portable digital scale), and fat and lean mass (EchoMRI-100^*TM*^, Echo Medical Systems, Houston, TX, USA), all female mice were crossed with male mice of the same strain and diet (day 0). After 2 days, all females were transferred to single housing for the duration of the study. Mice that did not become pregnant were considered non-pregnant controls. Echocardiography was performed on day 18, making the gestational time frame between day 15 and 17 of the 21-day murine gestational cycle (corresponding with third trimester in humans [9]).

### 2.2 Echocardiography

Echocardiography was performed as previously described [8,10] on isoflurane-anesthetized (2–4%, at effect, supplied via nose cone) pregnant and non-pregnant LF- and HF-fed female mice 18 days after being crossed with males. Briefly, images of the cross-sectional view of the LV at the papillary muscle-level in parasternal short-axis (PSAX) view were obtained in M-mode using an M550 transducer under the cardiology package on a Vevo 3100. Respiration rate and heart rate were monitored and adjusted to target 100 times/min and 400 beats/min, respectively, by titrating isoflurane levels. Images were analyzed with VevoLab Workstation software (VS-20034, Visual Sonics, Bothell, WA, USA) using LV trace methodology with data reported as mean of 3 cardiac cycles per mouse. The following parameters were measured over three cardiac cycles: thickness of the interventricular septum (IVS), LV interior diameter (LVID), and LV posterior wall (LVPW) and used to make the following calculations (via the VevoLab software): ejection fraction (EF; 100 × ((LV Vol; d – LV Vol; s) / LV Vol; d)), stroke volume (SV; LV Vol; d – LV Vol; s), LV mass (1.053 × (LVID; d + LVPW; d + IVS; d)3 – LVID; d3), and cardiac output (CO; SV × HR). Relative wall thickness (RTW) was calculated by 2* posterior wall thickness divided by LV diastolic diameter [11].

### 2.3 Statistical analyses

Data are presented as mean ± SEM. Statistical analyses of endpoints were performed using two-way analysis of variance (ANOVA) followed by Holm-Sidak for post hoc pairwise analysis in SigmaPlot (version 12.3, Systat, Palo Alto, CA, USA). All data passed normality or equal variance tests or logarithmic transformation was used to achieve normality. Values of *p <* 0.05 were considered to be statistically significant.

## 3. Results

Before pregnancy, mice fed a HF diet for 8 weeks had significantly increased body weight and fat mass, and decreased lean mass compared to LF-fed mice (*p <* 0.001, Supplementary Fig. 1A,B). In LF-fed mice, n = 10 mice became pregnant, and n = 20 mice were non-pregnant controls. In HF-fed mice, there were n = 12 pregnant and n = 18 non-pregnant mice. At the end of the study, third trimester of pregnancy, HF-fed females had significantly increased body weight compared to LF-fed controls (*p <* 0.001), and body weight was increased in pregnant compared to non-pregnant mice (*p <* 0.001, Supplementary Fig. 1C). However, there was no difference in body weight gain during pregnancy between LF and HF fed mice (either grams gained, or as percentage of body weight gained; Supplementary Fig. 1D).

**Fig. 1.**
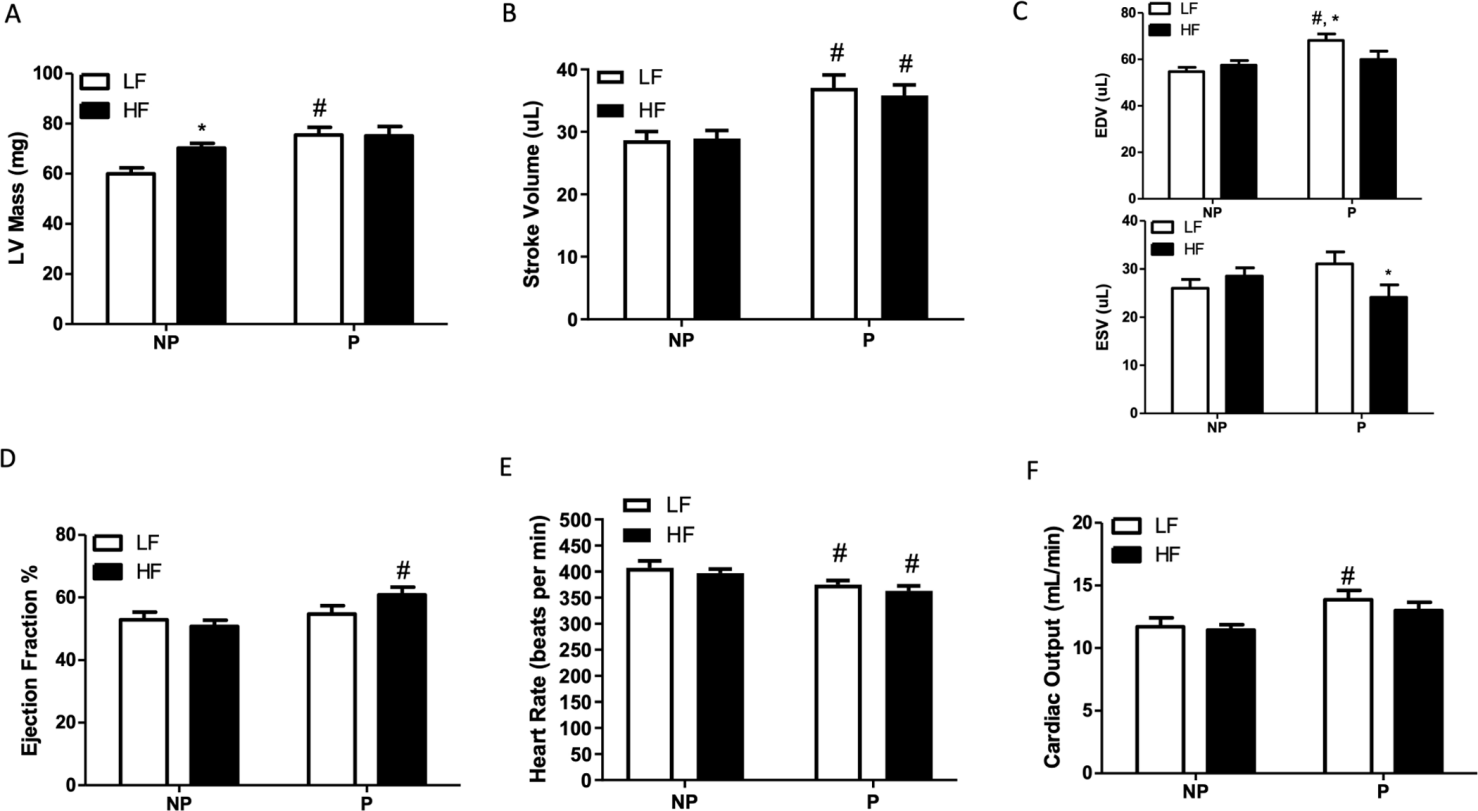
Cardiac adaptation to pregnancy is impaired in mice fed a high-fat diet. (A) LV mass. (B) Stroke volume. (C) End-diastolic volume (EDV) and end-systolic volume (ESV). (D) Ejection fraction. (E) Heart rate, and (F) cardiac output in pregnant (P) and non-pregnant (NP) female mice fed a low-fat (LF) or high-fat (HF) diet for 11 weeks. Data are mean + SEM from n = 20 LF NP, n = 10 LF P, n = 18 HF NP, and n = 12 HF P. *, *p <* 0.05 compared to LF; #, *p <* 0.05 compared to NP analyzed by 2-way ANOVA followed by Holm-Sidak pairwise analysis.

LV mass was increased with HF-feeding in non-pregnant mice (*p <* 0.05, Fig. 1A). However, only LF-fed mice exhibited cardiac hypertrophy with pregnancy (*p <* 0.001); LV mass was not augmented with pregnancy in HF-fed mice (*p* = 0.221, Fig. 1A). Both LF- and HF-fed mice displayed an increase in stroke volume (SV) with pregnancy (*p <* 0.001, Fig. 1B). Interestingly, elevated SV with pregnancy was associated with increased end-diastolic volume (EDV) in LF mice (*p <* 0.001), but not HF mice (Fig. 1C). Rather, compared to LF pregnant mice, HF pregnant mice exhibited a significantly decreased end-systolic volume (ESV, *p <* 0.05, Fig. 1C). Accordingly, ejection fraction (EF) was increased with pregnancy in HF (*p <* 0.01), but not LF mice (Fig. 1D). Heart rate of (isoflurane-anesthetized) pregnant mice was lower than non-pregnant mice with no effect of diet (*p <* 0.05, Fig. 1E), but cardiac output (CO) was significantly increased with pregnancy only in LF-mice (*p <* 0.01, Fig. 1F).

**Fig. 2.**
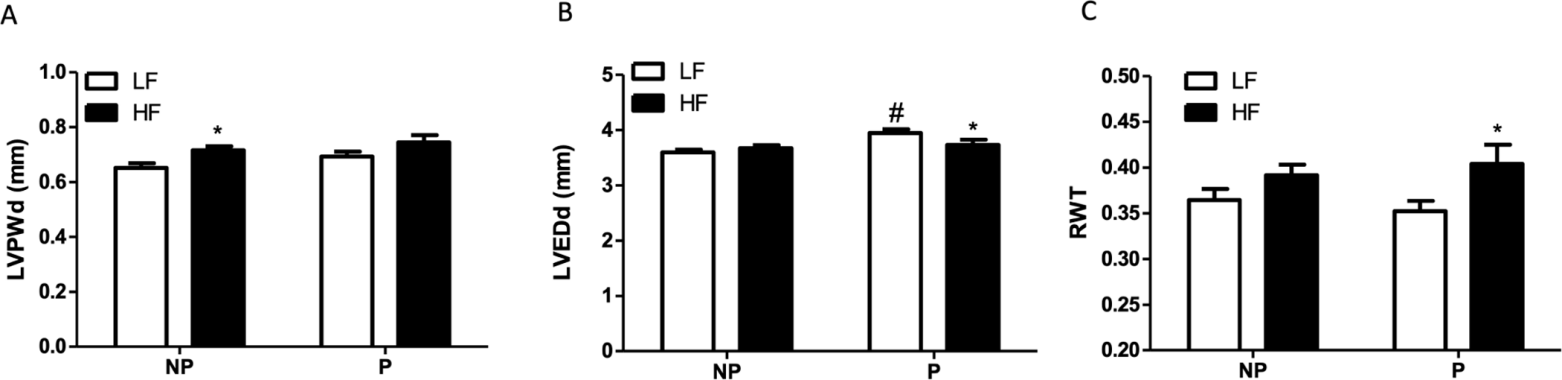
High fat feeding during pregnancy is associated with concentric LV remodeling. (A) Diameter of LV posterior wall (LVPWd). (B) Diameter of LV end-diastolic volume (LVEDd), and (C) relative wall thickness (RTW; 2 x posterior wall thickness divided by LV diastolic diameter) in pregnant (P) and non-pregnant (NP) female mice fed a low-fat (LF) or high-fat (HF) diet for 11 weeks. Data are mean + SEM from n = 20 LF NP, n = 10 LF P, n = 18 HF NP, and n = 12 HF P. *, *p <* 0.05 compared to LF; #, *p <* 0.05 compared to NP analyzed by 2-way ANOVA followed by Holm-Sidak pairwise analysis. Notably, LV chamber diameter is increased with pregnancy in LF-fed mice; in contrast, HF-fed mice exhibit increased RWT with pregnancy compared to LF-fed mice.

The increase in LV mass in non-pregnant HF-fed mice was associated with increased LV posterior wall thickness (*p <* 0.01, Fig. 2A). In contrast, increased LV mass with pregnancy in LF-fed mice was associated with an increase in the ventricle chamber (LV end-diastolic diameter [LVEDd], *p <* 0.001), with no change to the wall thickness, and the LVEDd was significantly larger in LF- versus HF-fed pregnant mice (*p <* 0.05, Fig. 2B). Thus, the RWT, a measure of LV geometry, was significantly increased in HF- compared to LF-fed mice during pregnancy (*p <* 0.05, Fig. 2C).

## 4. Discussion

Obesity promotes pathological cardiac hypertrophy, a prognostic indicator of CVD [12], and is generally associated with concentric remodeling. It is not well understood how physiological cardiac hypertrophy of pregnancy, tending to be eccentric in nature and assumed to be transient [13,14], is influenced by obesity. We previously demonstrated augmented cardiac hypertrophy postpartum in HF-fed mice compared to those that had never been pregnant [8]. Here, we report a differential effect of HF- versus LF-feeding during pregnancy on cardiac geometry in mice. The major findings from this study are: (1) LF-fed mice exhibited cardiac hypertrophy in response to pregnancy; while LV mass was increased with HF-feeding, it was not further augmented with pregnancy, (2) LF-fed mice with cardiac hypertrophy of pregnancy had increased LVED, but no change in wall thickness; in contrast, pregnant HF-fed mice had significantly smaller LVED and significantly greater RWT than pregnant LF-fed mice, and (3) increased SV with pregnancy was achieved via increased EDV in LF-fed mice, but decreased ESV in HF-fed mice. These findings suggest that cardiac remodeling in response to pregnancy was associated with an eccentric phenotype in lean mice (consistent with published literature in humans [14] and mice [7,15]), but a concentric phenotype in obese mice, where the expected physiologic remodeling was impaired. The significance of these findings is that obesity during pregnancy may promote pathological cardiac remodeling.

In a recent, similar study by Yang *et al*. [7], mice fed a Western (high sucrose, 45% kcal from fat) versus control diet for 15 weeks displayed increased LV mass and RWT during pregnancy associated with cardiomyocyte hypertrophy and fibrosis, and subsequent cardiac dysfunction postpartum in response to angiotensin II/phenylephrine challenge. The authors eloquently demonstrated in mice that pre-pregnancy risk (Western-diet induced metabolic syndrome) can lead to adverse cardiac effects during pregnancy (pathologic remodeling), and increased subsequent susceptibility to cardiac dysfunction. Findings from the current study extend those of Yang *et al*. [7] by demonstrating a similar cardiac morphologic phenotype in a different mouse model (very high fat diet of 60% kcal from fat), evidenced in a shorter time frame (8 weeks diet feeding prior to pregnancy versus 15 weeks). Interestingly, despite a markedly greater body weight and fat mass pre-pregnancy (32% and 284% increase in HF compared to LF, respectively), weight gain during pregnancy was nearly identical between LF- and HF-fed mice. This suggests that adverse effects of remodeling with HF feeding are not attributed to excess weight gain during pregnancy, but, in support with Yang *et al*. [7], are influenced by pre-existing health status (e.g., obesity or metabolic syndrome).

We previously demonstrated that 10 weeks after delivery, female mice fed a HFD displayed augmented cardiac hypertrophy and an altered expression profile of cardiac genes regulating hypertrophy and the extracellular matrix compared to HF-fed mice that had never been pregnant [8]. This is in agreement with Yang *et al*. [7], who also demonstrate a distinct gene expression profile in hearts from Western diet female mice. Taken together, this data supports the statement that cardiac remodeling in response to pregnancy is disrupted, likely creating conditions that promote increased risk for CVD postpartum.

LV diastolic dysfunction (more pronounced in women [16]) is a key indicator of heart failure with preserved ejection fraction (HFpEF), where female sex and BMI are strong risk factors [17]. Although variability in cardiac geometry exists, HFpEF is predominantly associated with increased concentricity [18]. Regiz-Zagrosek *et al*. [19] reported that while hearts of both men and women respond to pressure-overload with concentric remodeling, women tend to stay more in concentric hypertrophy compared to men, with greater preservation of indices of systolic function. There are limited (and conflicting) data describing cardiovascular function in obese versus lean pregnant women. Obesity during pregnancy is associated with increased blood pressure [5], no functional impairment [5], and moderate diastolic dysfunction [20]. Here, we report that both lean and obese mice exhibited increased SV with pregnancy (consistent with literature [21]). However, CO was only significantly increased with pregnancy in lean, and not obese mice, which is surprising, given the body weight difference. In contrast to lean mice, where EDV was increased, HF-fed mice had reduced ESV, which translated to elevated EF. It is a limitation of our work that we did not quantify diastolic function. However, given the increase in RWT in pregnant obese versus lean mice, it is logical assumption that hearts of obese pregnant mice exhibit impaired relaxation and/or increased stiffness. Pathologic effects of obesity during pregnancy may be a key component to increased susceptibility of women to LV diastolic dysfunction, and subsequent HFpEF.

## 5. Conclusions

In conclusion, we report that high fat feeding associated with increased body weight and fat mass disrupts physiologic cardiac remodeling in mice during pregnancy. This is in agreement with other studies demonstrating that pre-pregnancy cardiometabolic status promotes pathologic cardiac remodeling during pregnancy. We propose that detrimental effects of obesity on cardiac structure and function during the sensitive window of pregnancy may set the stage for subsequent CVD. This is important, as current cardiovascular risk assessment does not take pregnancy history into consideration. Future studies in mice and humans will define associations between cardiac changes during pregnancy with long-term cardiovascular health.

## Supplementary Material

Supplementary Material

Supplementary material

Supplementary material associated with this article can be found, in the online version, at 10.31083/j.rcm2301040.
