# Supplementary material for: Differential cardiac geometry during pregnancy in lean versus obese mice
Source: Rev Cardiovasc Med. Author manuscript; Available in PMC 2026 Aug 3. (PMC13431453; doi:10.31083/j.rcm2301040)
Supplement: Supplementary Material [file NIHMS2175275-supplement-Supplementary_Material.docx]

Supplementary material


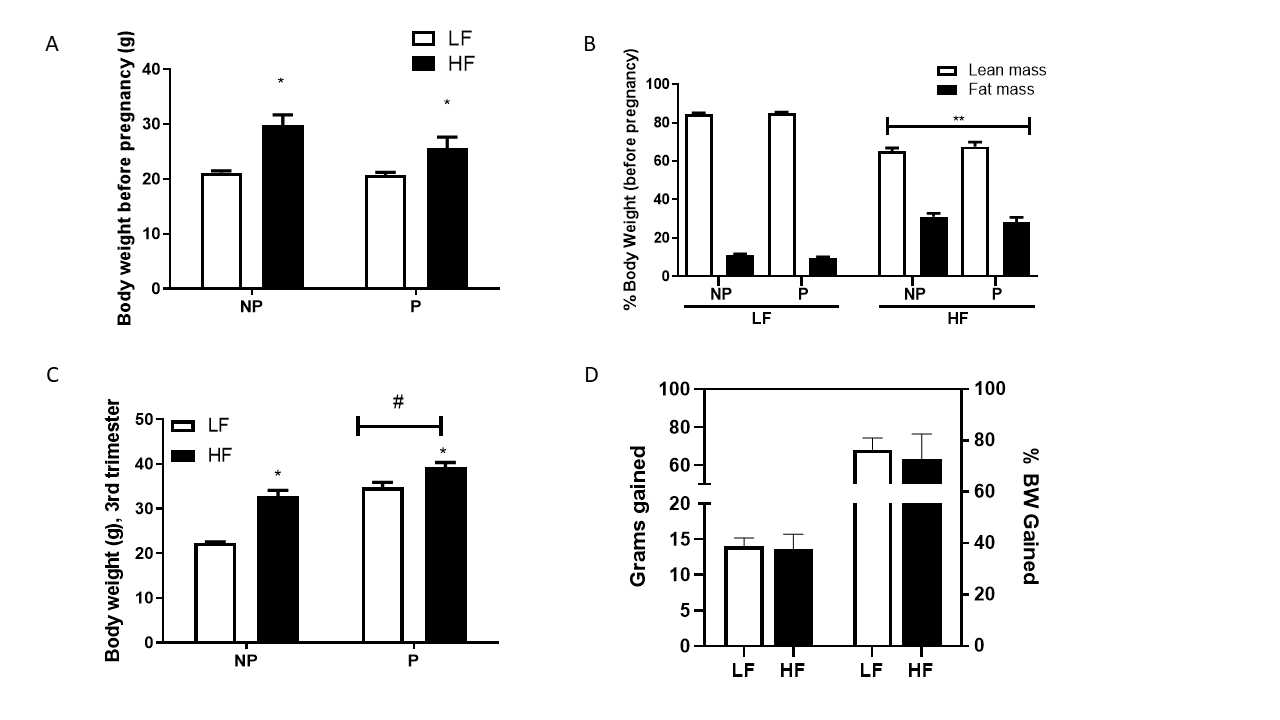


Supplementary Fig. S1. (A) Body weight and (B) lean and fat mass of mice fed a LF or HF diet for 8 weeks (before pregnancy). (C) Body weight of LF- and HF-fed pregnant mice at study endpoint. (D) Grams gained and % body weight gained during pregnancy in LF- and HF-fed mice. Data are mean + SEM in n = 10-20 mice per group. *, *p* < 0.001 effect of diet; #, *p* < 0.001 effect of pregnancy by 2-way ANOVA.
